# Medical Attendance on the Poor and the Public Health

**Published:** 1907-05-11

**Authors:** 


### PUBLIC HEALTH AND HYGIENE.

### MEDICAL ATTENDANCE ON THE POOR AND THE PUBLIC HEALTH.

IT is one of the misfortunes resulting from the diversity and intricacy of medical knowledge that medicine is divided into sections so numerous and varied that identity of interest becomes more and more difficult of discovery and description.

If there be any single aim, however, which may be said to be common to all departments of medicine it is the attainment of health. This constitutes a unity of purpose which links the diverse activities of medicine in a common bond, and the practitioner, the specialist, the laboratory investigator, and the administrative official may all alike be said to be engaged in pursuits which, while differing in detail, have one and the same end—the achievement and maintenance of health. Immediate aims often obscure, and even hide completely, the ultimate object of the pursuit, and in some such influence as this is to be found the cause of that division of interest which has grown apace with the ever-increasing specialisation of medical knowledge and practice. Nowhere has this separation of interest become more marked than in the gap which has come into existence between the provinces of preventive and curative medicine.

The separation of the medical officer of health from the family medical practitioner—a necessary stage in the development of public health administration—constitutes a divorce which is inimical to the solidarity of medicine. In essence every practitioner is, or should be, engaged in the conservation of the public health, and what is needed is an organic change in the conditions of medical practice that shall give administrative effect to that unity of purpose within the scope of which preventive and curative medicine are comprehended as merely convenient distinctions.

The problem of the adequate provision of medical attendance for the masses in this country has intruded itself, in fact, as a phase in the national question of the public health. It is seen that in the absence of any satisfactory solution of it there is great hardship to the sick and suffering who may be unable to secure that proper and skilled attendance which is so necessary to their state. But it is not so generally seen that, on the one hand, existing arrangements have failed to take the fullest advantage of that skill and knowledge of which the medical practitioner is the possessor, while, on the other hand, the national health is suffering grievously for lack of that very knowledge and skill which should be, but is not fully available for those who need it.

Thus it comes that we have a highly and expensively-trained profession labouring under conditions of practice which satisfy none, submitting with such appearance of good grace as the exigencies of life demand, but deeply, though for the most part silently, resentful of the unnecessary hardships and worries and of the inadequate rewards which are the practitioner's lot. Practice there is in abundance, if only it could be engaged in without regard to what we may, without offence, call the commercial needs of the practitioner. But because of these needs, and of the conditions they have called into being, years of restrained activity, of weary waiting for practice, are spent ere the hardly-won professional attainments can be exercised. Eventually come the trying responsibilities of practice, of gratuitous services to the poor rendered possible by those paid for by the well-to-do, of grudging requitals, or of undischarged and unacknowledged obligations. The public, for its part, out of its abundant charity, provides hospitals and dispensaries, homes, and agencies of medical relief of all descriptions, and what with medical practitioners and their clubs, hospitals and their out-departments, infirmaries and a Poor-law medical service, it thinks that the medical needs of the poor are amply provided. Yet, as every medical practitioner knows, this is not so. And if he, out of his comparatively meagre resources, did not contribute disproportionately to their relief, the poor—not necessarily the indigent—would be in much worse case. Yet, even so, there is probably more disease allowed perforce to go untreated than the amount which receives the attention of the medical man. We probably do not exaggerate when we say that there are more persons sick and requiring medical attention than all the patients privately attended, together with those treated in the hospitals, and provided for under the pauperising conditions of the Poor-law medical service.

Notwithstanding the complaints about an overstocked profession it is probably true that there is more medical work urgently in need of being performed than could properly be carried out by existing practitioners. It is not because there is not work to do that so many doctors must necessarily remain idle, so far as the practice of their profession is concerned. It is that the conditions of medical practice are inhibitive of services which it is equally the desire of the practitioners to grant and the patient to receive. The fact is that the economic condition of the millions does not permit of the system of fee-paying on which the general practitioner depends for the greater part of his income. The sudden and rarely anticipated demands of illness are a strain on the resources of those persons who compose the masses such as can only be met by organised provision. In so far as practitioners are dependent upon fees they cannot hope to be financially recompensed for their treatment of by far the greater part of afflicted humanity. On the other hand, privately organised provision in the shape of clubs with medical benefits, quite apart from its signal failure to embrace the masses below the fee-paying level, cannot be said to have dealt equitably with the practitioner. Such clubs have been looked at askance by the medical man and accepted only as a repugnant necessity to which he has submitted with the well-founded feeling that he was being ungenerously exploited. Where the income from private practice has been sufficient to leave the practitioner independent of contract work, this latter form of practice has usually been avoided.

This is not as it should be. Medicine has as its object the treatment of disease and the relief of suffering irrespective of the social status or financial position of the patient. Existing conditions of practice make well-nigh impossible the attainment of this object, and it is desirable alike in the interests of the public and of the rank and file of the profession that some more satisfactory organisation of the conditions of practice should be found. Nothing short of a civil medical service will meet the requirements of the situation. There is a growing feeling, if we mistake not, inside as well as outside the profession, in favour of this solution of the problem, and there is no question that this feeling will continue to grow as there is more full appreciation of the truth that, apart from humanitarian considerations, the economic advantages are vastly in its favour.

